# Correlation between traditional Chinese medicine constitution and occurrence of depressive symptoms in early pregnancy and early postpartum periods

**DOI:** 10.3389/fpsyt.2026.1750412

**Published:** 2026-07-28

**Authors:** Xiao-Wen Chen, Xu-Hong Zhu, Ying Chen, Li-Yuan Jiang

**Affiliations:** 1 Department of Obstetrics and Gynecology, Hangzhou Women’s Hospital (Hangzhou Maternity and Child Health Care Hospital), Hangzhou, Zhejiang, China; 2 Department of Women’s Health, Hangzhou Lin’an Qingshanhu Community Health Service Center, Hangzhou, Zhejiang, China

**Keywords:** balanced constitution, perinatal depressive symptoms, predictive role, Qi-stagnation, traditional Chinese medicine constitution

## Abstract

**Background:**

The impact of Traditional Chinese Medicine (TCM) constitution on predicting perinatal depressive symptoms remains underexplored, particularly regarding its longitudinal association with postpartum outcomes and the mediating effect of antenatal depressive symptoms.

**Objective:**

This study sought to examine the association between Traditional Chinese Medicine (TCM) constitution types during pregnancy and the risk of perinatal depressive symptoms, as well as to identify related influencing factors.

**Methods:**

A prospective cohort study was conducted, involving pregnant women at 10–14 weeks of gestation (n=1286). The Classification and Determination of Constitution in Traditional Chinese Medicine self-assessment form was employed to evaluate constitution types, while the Edinburgh Postnatal Depression Scale (EPDS) was utilized to assess depressive status during both pregnancy and the postpartum period. Additionally, pertinent demographic and obstetric characteristic data were collected. Mediation analysis with bootstrap resampling (5,000 iterations) was performed to examine indirect pathways.

**Results:**

The findings revealed that the qi-stagnation constitution was significantly associated with an increased risk of prenatal depressive symptoms (OR = 2.58, 95% CI: 1.59-4.19, P<0.001), whereas the balanced constitution was linked to a decreased risk of prenatal depressive symptoms (OR = 0.35, 95% CI: 0.24-0.51, P<0.001).After adjusting for antenatal depressive symptoms, the qi-stagnation constitution (OR = 2.04, 95% CI: 1.14–3.67, P = 0.017) and preterm birth before 34 weeks of gestation (OR = 4.26, 95% CI: 1.45–12.47, P = 0.008)have been identified as independent risk factors for postpartum depressive symptoms. Conversely, the balanced constitution demonstrates a protective effect(OR = 0.42, 95% CI: 0.26–0.68, P < 0.001). Additionally, prenatal depressive symptoms significantly elevates the risk of postpartum depressive symptoms (OR = 2.05, 95% CI: 1.35–3.09, P < 0.001).and significantly mediated the association between Qi-stagnation constitution and postpartum depressive symptoms (indirect effect = 0.36, 95% Bootstrap CI: 0.19–0.57).

**Conclusion:**

In summary, the qi-stagnation constitution emerges as a central risk factor for perinatal depressive symptoms, whereas the balanced constitution serves as a protective factor. Preterm birth and prenatal depressive symptoms are associated with an increased risk of postpartum depressive symptoms. These findings support integrating TCM constitution identification with depressive symptoms screening during early pregnancy. For women identified with a qi-stagnation constitution, a comprehensive strategy involving ‘constitution regulation and psychological intervention’ should be implemented to reduce the incidence of perinatal depressive symptoms. Further research should evaluate the effectiveness of these interventions in randomized controlled trials.

## 1 Introduction

Perinatal depressive symptoms —a syndrome involving low mood or loss of interest in pregnancy or for up to one year after birth—occurs globally with an approximate prevalence of 7–13% (1). Within China, studies so far have noted somewhat lower rates, varying between 3. 8%and 16. 7% (2). This condition poses a substantial threat to maternal physical and mental health (3), and is closely associated with various long-term adverse outcomes, including delayed cognitive development in offspring and deteriorating family relationships (4, 5). The etiology of perinatal depressive symptoms has been linked to genetic, hormonal, and psychosocial causes, but now more attention is being given to individual differences in constitution as possible determinants of vulnerability to the disorder (6).

In Traditional Chinese Medicine (TCM) theory, constitution refers to an individual’s relatively stable physiological and psychological characteristics, shaped by both innate endowment and acquired factors such as diet, lifestyle, emotional state, and environment (1). Importantly, constitution is not fixed; it can shift over time in response to changes in daily habits, emotional health, and overall balance (7, 8). The nine constitutional types are classified based on a standardized instrument developed by the China Association of Chinese Medicine (9). Among these, the Balanced constitution represents an optimal state of physical and psychological harmony9. In contrast, biased constitutions—such as Qi-deficiency, Yang-deficiency, Yin-deficiency, Phlegm-Dampness, Dampness-Heat, Blood-Stasis, Qi-stagnation, and Inherited-Special—reflect various imbalances that may predispose individuals to specific health conditions (7). The Qi-stagnation constitution, in particular, is characterized by constrained flow of Qi (vital energy), often manifesting as emotional lability, a tendency toward rumination, and heightened stress reactivity (9).

Recent studies have linked specific TCM constitutions to depressive symptoms. Qi-stagnation has been found to be strongly associated with emotional disturbance and depressive symptoms (10), while Yang-deficiency appears to accompany both anxiety and depression in allergic rhinitis sufferers (11). However, the existing literature has been mostly devoted to the link between postpartum constitution and depression (12), with little attention to the longitudinal relationship between prenatal constitution and mental health—or to its predictive value for later postpartum states. In addition, the moderating roles of obstetric characteristics—such as parity or mode of delivery—in the constitution–depression relationship remain unclear. Furthermore, a growing body of longitudinal evidence has established that antenatal depressive symptoms are among the strongest predictors of postpartum depression (13). However, the extent to which individual constitutional factors modify or mediate this relationship remains largely unexplored.

To address these gaps, this prospective cohort study was designed with three primary objectives: (1) to examine the extent to which prenatal TCM constitution predicts the risk of perinatal depressive symptoms; (2) to investigate the interaction effects between TCM constitution and obstetric factors; and (3) to characterize the dynamic changes in depressive states over time.

## 2 Methods

### 2.1 Study participants and data collection

The research involved pregnant women between 10 and 14 weeks of gestation who were registered at community health centers in Hangzhou (n=1286). The inclusion criteria comprised: (1) singleton pregnancy; (2) age ≥ 18 years; (3) ability to read and understand Chinese; (4) absence of prior somatic or psychiatric disorders; (5) voluntary participation with written informed consent; and (6) absence of severe pregnancy complications such as preeclampsia or placental abruption. Exclusion criteria encompassed thyroid dysfunction, a history of psychiatric disorders prior to pregnancy, severe diseases such as epilepsy or tumors, fetal death, and loss to follow-up. A history of psychiatric disorders, including depression, prior to pregnancy was excluded through a two-step process: (1) an initial self-report during the screening interview, and (2) subsequent verification against electronic medical records from the Hangzhou Maternal and Child Health Information System, which provides access to patients’ prior medical visit records. All researchers involved in data collection and medical record review received standardized training to ensure consistency. Data were collected on several parameters, including demographic and obstetric characteristics such as age, gravidity, parity, mode of delivery (vaginal or cesarean), and gestational age at delivery. In this cohort, all participants were married with living spouses. The classification of Traditional Chinese Medicine (TCM) constitution was evaluated between 10 and 14 weeks of gestation using the Classification and Determination of TCM Constitution Self-Assessment Scale, as per the standards of the China Association of Chinese Medicine (9), This assessment categorized participants into nine types, including Balanced, Qi-deficiency, and Qi-stagnation, with constitution bias determined through conversion scores. Depressive symptoms were assessed using the Edinburgh Postnatal Depression Scale (EPDS) both antenatally, at 10 to 14 weeks, and within 14 days postpartum. Follow-up data on neonatal outcomes, including height, weight, sex, and birth defects, were obtained from electronic medical records and supplemented by telephone follow-up.

### 2.2 Assessments

Two assessment instruments were used in this study: the TCM Constitution Classification and the Edinburgh Postnatal Depression Scale (EPDS).

#### 2.2.1 TCM constitution classification

The TCM constitution classification is based on a standardized instrument developed by the China Association of Chinese Medicine. Constitution types were identified according to the guidelines set forth in the *Classification and Determination of TCM Constitution (ZYYXH/T157-2009) (*9). A Balanced constitution was characterized by a conversion score of ≥60, with all other types scoring <30. For biased constitutions, scores of ≥40 indicated a definitive bias, while scores ranging from 30 to 39 suggested a tendency towards bias. In cases where multiple biases met the criteria, the type with the highest score was selected (14).

#### 2.2.2 Edinburgh postnatal depression scale

The Edinburgh Postnatal Depression Scale (EPDS), a 10-item instrument with a score range of 0–30, was utilized to evaluate antenatal and postpartum depressive symptoms (15). The EPDS is a screening instrument rather than a diagnostic tool; it is designed to identify individuals at risk of perinatal depression who may require further clinical assessment. The Chinese version of the EPDS has demonstrated high reliability and validity. According to expert consensus, a score of ≥9 on the EPDS was indicative of perinatal depression (16).

### 2.3 Data management and statistical analysis

All researchers underwent standardized training to ensure methodological consistency. Data analysis was conducted using SPSS version 26.0. Continuous variables with a normal distribution were reported as mean ± standard deviation and analyzed using independent t-tests or one-way ANOVA. Non-normally distributed variables were presented as median (interquartile range) and analyzed using non-parametric tests. Categorical variables were expressed as frequencies and percentages (n [%]) and were compared using χ² tests or Fisher’s exact tests. For descriptive analyses, participants were divided into two groups based on the EPDS cutoff of ≥ 9 (presence of depressive symptoms) versus < 9 (absence), and baseline characteristics were compared between the two groups. A multivariable logistic regression analysis, adjusted for potential confounders such as age, occupation, education, income, gravidity, parity, delivery mode, and gestational age, was conducted to assess the independent associations between Traditional Chinese Medicine (TCM) constitution and perinatal depressive symptoms. The findings were expressed as odds ratios (OR) with 95% confidence intervals (CI), with statistical significance determined at an alpha level of 0.05 (P<0.05). To formally examine the mediating role of antenatal depressive symptom severity in the relationship between Qi-stagnation constitution and postpartum depressive symptoms, a mediation analysis was performed using the PROCESS macro (Model 4) for SPSS. In this model, the mediator (M) was the continuous EPDS score measured at 10–14 weeks of gestation, rather than a dichotomized depression status. The significance of the indirect effect was tested using a bootstrap resampling procedure with 5,000 iterations. An indirect effect was considered statistically significant if the 95% bootstrap confidence interval (CI) for the indirect effect did not include zero. Because the outcome variable (postpartum depressive symptoms) was dichotomous, the mediation model was estimated using logistic regression for the outcome equation, and all effects are reported in log-odds units.

### 2.4 Ethical considerations

The study received approval from the hospital ethics committee (Approval No. 2021A(7)02), and all participants provided informed consent. Data were anonymized to ensure confidentiality.

## 3.Results

The final analysis included 1,286 participants who completed both antenatal and postpartum assessments. 214 participants, accounting for 14.27%, were lost to follow-up during the postpartum period and were not included in the analysis. The mean maternal age was 31.0 years (IQR: 28.0–34.0), and the median gestational age at delivery was 39.0 weeks (IQR: 38.0–39.75). Among the participants, 646 (50.2%) were local residents, 799 (62.1%) were primiparous, and 671 (52.2%) delivered vaginally. The prevalence of antenatal depressive symptoms (EPDS ≥ 9) was 18.7% (n = 241), and the prevalence of postpartum depressive symptoms was 10.0% (n = 129).

### 3.1 Association between prenatal TCM constitution and antenatal depressive symptoms

Univariate analysis (**Table 1**) showed significant associations between Qi-stagnation constitution (χ² = 31.98, P < 0.001), Balanced constitution (χ² = 55.52, P < 0.001), and Qi-deficiency constitution (χ² = 10.48, P = 0.001) with antenatal depressive symptoms (EPDS ≥ 9). Other TCM constitutions and obstetric variables, including age, gravidity, parity, residence, and scarred uterus, showed no statistical significance (all P > 0.05).

**Table 1:** Univariate analysis of the relationship between TCM constitution and antenatal depressive symptoms (EPDS at ≤13 weeks’ gestation, cut-off ≥9).

Variables	Total (n = 1286)	EPDS <9 (n=1,045) (n = 1045)	EPDS ≥9 (n = 241)	Statistic	*P*
Maternal age, yrs, M (Q_1_, Q_3_)	31.00 (28.00, 34.00)	31.00 (28.00, 34.00)	30.00 (28.00, 34.00)	Z=-0.59	0.553
Gestational age at delivery, wks, M (Q_1_, Q_3_)	39.00 (38.00, 39.75)	39.00 (38.00, 40.00)	39.00 (38.00, 39.00)	Z=-0.43	0.665
Residence, n (%)				χ²=0.08	0.782
Local	646 (50.23)	523 (50.05)	123 (51.04)		
Non-local	640 (49.77)	522 (49.95)	118 (48.96)		
Gravidity, n (%)				χ²=0.83	0.660
1	584 (45.41)	478 (45.74)	106 (43.98)		
2	359 (27.92)	286 (27.37)	73 (30.29)		
≥3	343 (26.67)	281 (26.89)	62 (25.73)		
Parity, n (%)				χ²=0.13	0.937
Primipara	799 (62.13)	647 (61.91)	152 (63.07)		
2	408 (31.73)	333 (31.87)	75 (31.12)		
≥3	79 (6.14)	65 (6.22)	14 (5.81)		
Age, n (%)				χ²=0.66	0.415
<35	1043 (81.10)	852 (81.53)	191 (79.25)		
≥35	243 (18.90)	193 (18.47)	50 (20.75)		
Yang-deficiency, n (%)				χ²=2.88	0.090
No	1149 (89.35)	941 (90.05)	208 (86.31)		
Yes	137 (10.65)	104 (9.95)	33 (13.69)		
Yin-deficiency, n (%)				χ²=0.22	0.636
No	1197 (93.08)	971 (92.92)	226 (93.78)		
Yes	89 (6.92)	74 (7.08)	15 (6.22)		
Qi-deficiency, n (%)				χ²=10.48	**0.001**
No	1108 (86.16)	916 (87.66)	192 (79.67)		
Yes	178 (13.84)	129 (12.34)	49 (20.33)		
Phlegm-dampness, n (%)				χ²=0.28	0.597
No	1220 (94.87)	993 (95.02)	227 (94.19)		
Yes	66 (5.13)	52 (4.98)	14 (5.81)		
Dampness-heat, n (%)				χ²=1.72	0.190
No	1136 (88.34)	929 (88.90)	207 (85.89)		
Yes	150 (11.66)	116 (11.10)	34 (14.11)		
Blood-stasis, n (%)				χ²=0.21	0.645
No	1254 (97.51)	1020 (97.61)	234 (97.10)		
Yes	32 (2.49)	25 (2.39)	7 (2.90)		
Inherited-special, n (%)				χ²=1.02	0.312
No	1266 (98.44)	1031 (98.66)	235 (97.51)		
Yes	20 (1.56)	14 (1.34)	6 (2.49)		
Qi-stagnation, n (%)				χ²=31.98	**<.001**
No	1203 (93.55)	997 (95.41)	206 (85.48)		
Yes	83 (6.45)	48 (4.59)	35 (14.52)		
Balanced, n (%)				χ²=55.52	**<.001**
No	756 (58.79)	563 (53.88)	193 (80.08)		
Yes	530 (41.21)	482 (46.12)	48 (19.92)		
Scarred uterus				χ²=0.00	0.983
No	1088 (84.60)	884 (84.59)	204 (84.65)		
Yes	198 (15.40)	161 (15.41)	37 (15.35)		

Z: Mann-Whitney test, χ²: Chi-square test M: Median, Q_1_: 1st Quartile, Q_3_: 3rd Quartile.

Bold values indicate statistical significance (P < 0.05).

Multivariable logistic regression (**Table 2**), adjusting for potential confounders, confirmed that Qi-stagnation (P < 0.001, OR = 2.58, 95% CI: 1.59–4.19) and Balanced constitution (P < 0.001, OR = 0.35, 95% CI: 0.24–0.51) remained independent predictors of antenatal depressive symptoms, while Qi-deficiency did not retain statistical significance (P = 0.138, OR = 1.35, 95% CI: 0.91–1.99).

**Table 2:** Multivariate analysis of the relationship between traditional Chinese medicine (TCM) constitution and antenatal depressive symptoms (variables with P < 0.05 in univariate analysis were included).

Variables	β	S.E	Z	P	OR (95%CI)
Intercept	-1.26	0.11	-11.66	**<.001**	0.28 (0.23 ~ 0.35)
Qi-deficiency, n (%)					
No					1.00 (Reference)
Yes	0.30	0.20	1.48	0.138	1.35 (0.91 ~ 1.99)
Qi-stagnation, n (%)					
No					1.00 (Reference)
Yes	0.95	0.25	3.84	**<.001**	2.58 (1.59 ~ 4.19)
Balanced, n (%)					
No					1.00 (Reference)
Yes	-1.04	0.19	-5.60	**<.001**	0.35 (0.24 ~ 0.51)

OR, Odds Ratio; CI, Confidence Interval.

Bold values indicate statistical significance (P < 0.05).

### 3.2 TCM constitution and postpartum depressive symptoms

Univariate analysis (**Table 3**) revealed that significant predictors of postpartum depressive symptoms included Qi-stagnation (χ² = 16.26, P < 0.001), Balanced constitution (χ² = 26.24, P < 0.001), gravidity (χ² = 8.43, P = 0.015), parity (χ² = 9.51, P = 0.009), gestational age less than 34 weeks (χ² = 4.56, P = 0.010), antepartum EPDS score as a continuous variable (Z = -5.70, P < 0.001), and antenatal EPDS ≥ 9 (χ² = 24.54, P < 0.001). Blood-stasis showed a borderline association (χ² = 3.84, P = 0.050), and Qi-deficiency approached borderline significance (χ² = 3.69, P = 0.055). Other variables, including age, residence, mode of delivery, fetal sex, birth defects, antenatal anemia, gestational diabetes, gestational hypertension, fetal distress, fetal growth restriction, scarred uterus, psychological interventions, and most TCM constitutions (e.g., Yin-deficiency, Phlegm-dampness) showed no statistical significance (all P > 0.05).

**Table 3:** Univariate analysis of the relationship between traditional Chinese medicine (TCM) constitution and postpartum depressive symptoms (EPDS within 14 days postpartum, cut-off ≥9).

Variables	Total (n = 1286)	EPDS <9 (n=1157)	EPDS ≥9 (n =129)	Statistic	P
Maternal age, yrs, M (Q_1_, Q_3_)	31.00 (28.00, 34.00)	31.00 (28.00, 34.00)	30.00 (28.00, 33.00)	Z=-0.90	0.368
Gestational age at delivery, wks, M (Q_1_, Q_3_)	39.00 (38.00, 39.75)	39.00 (38.00, 40.00)	39.00 (38.00, 39.00)	Z=-0.74	0.460
Antepartum EPDS score, M (Q_1_, Q_3_)	5.00 (3.00, 8.00)	5.00 (3.00, 8.00)	7.00 (5.00, 10.00)	Z=-5.70	**<.001**
Residence, n (%)				χ²=0.00	0.971
Local	646 (50.23)	581 (50.22)	65 (50.39)		
Non-local	640 (49.77)	576 (49.78)	64 (49.61)		
Gravidity, n (%)				χ²=8.43	**0.015**
1	584 (45.41)	511 (44.17)	73 (56.59)		
2	359 (27.92)	326 (28.18)	33 (25.58)		
≥3	343 (26.67)	320 (27.66)	23 (17.83)		
Parity, n (%)				χ²=9.51	**0.009**
Primipara	799 (62.13)	703 (60.76)	96 (74.42)		
2	408 (31.73)	379 (32.76)	29 (22.48)		
≥3	79 (6.14)	75 (6.48)	4 (3.10)		
Age, n (%)				χ²=0.01	0.929
<35	1043 (81.10)	938 (81.07)	105 (81.40)		
≥35	243 (18.90)	219 (18.93)	24 (18.60)		
Gestational age at delivery, n (%)				χ²=4.56	0.102
≥37	1190 (92.53)	1074 (92.83)	116 (89.92)		
34-36	75 (5.83)	67 (5.79)	8 (6.20)		
<34	21 (1.63)	16 (1.38)	5 (3.88)		
Mode of delivery, n (%)				χ²=2.38	0.123
Vaginal delivery	671 (52.18)	612 (52.90)	59 (45.74)		
Cesarean section	615 (47.82)	545 (47.10)	70 (54.26)		
Antenatal anemia, n (%)				χ²=0.80	0.669
None	1087 (84.53)	975 (84.27)	112 (86.82)		
Mild	154 (11.98)	140 (12.10)	14 (10.85)		
Moderate/Severe	45 (3.50)	42 (3.63)	3 (2.33)		
Antenatal EPDS score, n(%)				χ²=24.54	**<.001**
<9	1045 (81.26)	961 (83.06)	84 (65.12)		
≥9	241 (18.74)	196 (16.94)	45 (34.88)		
Fetal sex, n (%)				χ²=1.24	0.265
Female	638 (49.61)	568 (49.09)	70 (54.26)		
Male	648 (50.39)	589 (50.91)	59 (45.74)		
Birth defects, n (%)				χ²=0.33	0.564
No	1251 (97.28)	1124 (97.15)	127 (98.45)		
Yes	35 (2.72)	33 (2.85)	2 (1.55)		
Yang-deficiency, n (%)				χ²=0.96	0.327
No	1149 (89.35)	1037 (89.63)	112 (86.82)		
Yes	137 (10.65)	120 (10.37)	17 (13.18)		
Yin-deficiency, n (%)				χ²=0.15	0.695
No	1197 (93.08)	1078 (93.17)	119 (92.25)		
Yes	89 (6.92)	79 (6.83)	10 (7.75)		
Qi-deficiency, n (%)				χ²=3.69	0.055
No	1108 (86.16)	1004 (86.78)	104 (80.62)		
Yes	178 (13.84)	153 (13.22)	25 (19.38)		
Phlegm-dampness, n (%)				χ²=0.34	0.562
No	1220 (94.87)	1099 (94.99)	121 (93.80)		
Yes	66 (5.13)	58 (5.01)	8 (6.20)		
Dampness-heat, n (%)				χ²=0.32	0.572
No	1136 (88.34)	1024 (88.50)	112 (86.82)		
Yes	150 (11.66)	133 (11.50)	17 (13.18)		
Blood-stasis, n (%)				χ²=3.84	0.050
No	1254 (97.51)	1132 (97.84)	122 (94.57)		
Yes	32 (2.49)	25 (2.16)	7 (5.43)		
Inherited-special, n (%)				χ²=1.28	0.259
No	1266 (98.44)	1137 (98.27)	129 (100.00)		
Yes	20 (1.56)	20 (1.73)	0 (0.00)		
Qi-stagnation, n (%)				χ²=16.26	**<.001**
No	1203 (93.55)	1093 (94.47)	110 (85.27)		
Yes	83 (6.45)	64 (5.53)	19 (14.73)		
Balanced, n (%)				χ²=26.24	**<.001**
No	756 (58.79)	653 (56.44)	103 (79.84)		
Yes	530 (41.21)	504 (43.56)	26 (20.16)		
Gestational diabetes, n (%)				χ²=2.43	0.119
No	1105 (85.93)	1000 (86.43)	105 (81.40)		
Yes	181 (14.07)	157 (13.57)	24 (18.60)		
Gestational hypertension, n (%)				χ²=0.00	1.000
No	1260 (97.98)	1134 (98.01)	126 (97.67)		
Yes	26 (2.02)	23 (1.99)	3 (2.33)		
Fetal distress, n (%)				χ²=0.01	0.930
No	1270 (98.76)	1142 (98.70)	128 (99.22)		
Yes	16 (1.24)	15 (1.30)	1 (0.78)		
Fetal growth restriction, n (%)				χ²=0.00	1.000
No	1273 (98.99)	1145 (98.96)	128 (99.22)		
Yes	13 (1.01)	12 (1.04)	1 (0.78)		
Scarred uterus				χ²=1.56	0.211
No	1088 (84.60)	974 (84.18)	114 (88.37)		
Yes	198 (15.40)	183 (15.82)	15 (11.63)		
Psychological intervention, n (%)				χ²=0.00	0.950
No	1250 (97.20)	1124 (97.15)	126 (97.67)		
Yes	36 (2.80)	33 (2.85)	3 (2.33)		

Z: Mann-Whitney test, χ²: Chi-square test.

Bold values indicate statistical significance (P < 0.05).

Multivariable logistic regression (Model 1, without antenatal EPDS, **Table 4**), including gravidity, parity, and gestational age, confirmed that Qi-stagnation (P = 0.003, OR = 2.36, 95% CI: 1.33–4.19), Balanced constitution (P < 0.001, OR = 0.38, 95% CI: 0.24–0.61), and gestational age under 34 weeks (P = 0.007, OR = 4.39, 95% CI: 1.49–12.97) remained independent risk factors, while blood-stasis did not retain significance (P = 0.103, OR = 2.08, 95% CI: 0.86–5.00).

**Table 4:** Multivariate analysis of the relationship between traditional Chinese medicine (TCM) constitution and postpartum depressive symptoms – Model 1(without antenatal EPDS).

Variables	β	S.E	Z	P	OR (95%CI)
Intercept	-1.82	0.15	-12.36	**<.001**	0.16 (0.12 ~ 0.22)
Parity times, n (%)					
1					1.00 (Reference)
2	-0.17	0.27	-0.64	0.521	0.84 (0.50 ~ 1.43)
≥3	-0.34	0.36	-0.95	0.341	0.71 (0.35 ~ 1.44)
Parity, n (%)					
Primipara					1.00 (Reference)
2	-0.30	0.31	-0.97	0.333	0.74 (0.40 ~ 1.36)
≥3	-0.60	0.63	-0.96	0.335	0.55 (0.16 ~ 1.87)
Blood-stasis					
No					1.00 (Reference)
Yes	0.73	0.45	1.63	0.103	2.08 (0.86 ~ 5.00)
Qi-stagnation					
No					1.00 (Reference)
Yes	0.86	0.29	2.92	**0.003**	2.36 (1.33 ~ 4.19)
Balanced					
No					1.00 (Reference)
Yes	-0.97	0.24	-4.04	**<.001**	0.38 (0.24 ~ 0.61)
Gestational age at delivery, n (%)					
≥37					1.00 (Reference)
34-36	0.20	0.40	0.51	0.609	1.23 (0.56 ~ 2.67)
<34	1.48	0.55	2.68	**0.007**	4.39 (1.49 ~ 12.97)

OR, Odds Ratio; CI, Confidence Interval.

Bold values indicate statistical significance (P < 0.05).

When antenatal depressive symptoms (EPDS ≥ 9) were added to the model (Model 2, **Table 5**), they significantly elevated the risk of postpartum depressive symptoms (P < 0.001, OR = 2.05, 95% CI: 1.35–3.09). After adjusting for antenatal depressive symptoms, Qi-stagnation remained significant (P = 0.017, OR = 2.04, 95% CI: 1.14–3.67), Balanced constitution remained protective (P < 0.001, OR = 0.42, 95% CI: 0.26–0.68), and gestational age under 34 weeks also remained significant (P = 0.008, OR = 4.26, 95% CI: 1.45–12.47). Parity was included as a covariate in the regression models because, although all participants had singleton pregnancies, the number of previous births may influence both psychological adjustment during pregnancy and postpartum recovery.

**Table 5:** Multivariate analysis of the relationship between traditional Chinese medicine (TCM) constitution and postpartum depressive symptoms– Model 2(Antenatal EPDS ≥9 added).

Variables	β	S.E	Z	P	OR (95%CI)
Intercept	-2.01	0.16	-12.35	**<.001**	0.13 (0.10 ~ 0.18)
Parity times, n (%)					
1					1.00 (Reference)
2	-0.20	0.27	-0.74	0.456	0.82 (0.48 ~ 1.39)
≥3	-0.36	0.36	-0.98	0.326	0.70 (0.34 ~ 1.43)
Parity, n (%)					
Primipara					1.00 (Reference)
2	-0.30	0.31	-0.95	0.344	0.74 (0.40 ~ 1.38)
≥3	-0.60	0.63	-0.96	0.337	0.55 (0.16 ~ 1.88)
Blood-stasis					
No					1.00 (Reference)
Yes	0.74	0.45	1.63	0.104	2.09 (0.86 ~ 5.10)
Qi-stagnation					
No					1.00 (Reference)
Yes	0.71	0.30	2.39	**0.017**	2.04 (1.14 ~ 3.67)
Balanced					
No					1.00 (Reference)
Yes	-0.86	0.24	-3.51	**<.001**	0.42 (0.26 ~ 0.68)
Gestational age at delivery, n (%)					
≥37					1.00 (Reference)
34-36	0.22	0.40	0.56	0.574	1.25 (0.57 ~ 2.73)
<34	1.45	0.55	2.64	**0.008**	4.26 (1.45 ~ 12.47)
Antenatal EPDS score					
<9					1.00 (Reference)
≥9	0.72	0.21	3.39	**<.001**	2.05 (1.35 ~ 3.09)

OR, Odds Ratio; CI, Confidence Interval.

Bold values indicate statistical significance (P < 0.05).

### 3.3 Mediating role of antenatal EPDS score

To formally test whether antenatal depressive symptom severity mediated the association between Qi-stagnation constitution and postpartum depressive symptoms, we conducted a mediation analysis using the PROCESS macro (Model 4) with 5,000 bootstrap resamples. In this model, the mediator was the continuous EPDS score measured at 10–14 weeks of gestation, and the outcome was postpartum depressive symptoms (dichotomized at EPDS≥9). Mediation analysis revealed that Qi-stagnation constitution significantly predicted higher antenatal EPDS scores (a = 2.80, SE = 0.41, P < 0.001), and higher antenatal EPDS scores significantly predicted an increased risk of postpartum depressive symptoms (b = 0.13, SE = 0.02, P < 0.001). The indirect effect of Qi-stagnation constitution on postpartum depressive symptoms via antenatal EPDS scores was significant (ab = 0.36, BootSE = 0.10, 95% Bootstrap CI: 0.19 to 0.57). Because the 95% confidence interval did not include zero, the mediating effect was statistically confirmed. After accounting for the indirect pathway, the direct effect of Qi-stagnation constitution on postpartum depressive symptoms remained significant (c’ = 0.73, SE = 0.30, P = 0.014). This pattern indicates that antenatal depressive symptom severity partially mediated the relationship between Qi-stagnation constitution and postpartum depressive symptoms, consistent with the attenuation of the odds ratio observed in the multivariable logistic regression models (from OR = 2.36 to OR = 2.04 when antenatal EPDS≥9 was added).

## 4 Discussion

This prospective cohort study provides evidence that Qi-stagnation and Balanced constitutions are independent predictors of both antenatal and postpartum depressive symptoms. Additionally, it has been demonstrated that a gestational age of less than 34 weeks increases the risk of postpartum depressive symptoms. Mediation analysis using bootstrap resampling further confirmed that antenatal depressive symptoms partially mediate the relationship between Traditional Chinese Medicine (TCM) constitution and postpartum depressive symptoms.

### 4.1 Association between TCM constitution and prenatal depressive symptoms

The analysis shows clearly that Qi - stagnation is strongly linked to an increased risk of prenatal depressive symptoms (OR = 2.58, P<0.001),while a Balanced constitution seems to be protective (OR = 0.35, P<0.001). Such conclusions correspond well with ideas in traditional Chinese medicine about constitution and emotion. Qi-stagnation whether arising from emotional or physical factors, is intimately tied to depressive symptoms. Earlier research has indicated that people suffering from Qi - stagnation tend to be more likely than others to experience low mood or depression under stress or in emotional difficulty (17). Another study gives further support to the idea that Qi - stagnation is connected to a vulnerability to mental ailments such as anxiety or depression (11).In contrast, the Balanced constitution is recognized as ideal by TCM (Traditional Chinese Medicine). Compared to other constitutions, Balanced constitution individuals have fewer psychological issues like depression and anxiety and stronger psychological adaptability (18). Such findings indicate that prenatal care needs to be directed toward pregnant women with a tendency to Qi - stagnation, with early psychological treatment and constitution - related conditioning to cut down depression rates.

### 4.2 Association between TCM constitution and postpartum depressive symptoms

This study identified preterm delivery (<34 weeks), Qi-stagnation and Balanced constitutions, and prenatal depressive symptoms as independent risk factors for postpartum depressive symptoms. Qi-stagnation - prone women had a significantly higher postpartum depressive symptoms risk (OR = 2.36, 95% CI: 1.33–4.19, P = 0.003). The mediating role of antenatal depressive symptom severity was formally confirmed through bootstrap mediation analysis. The indirect effect of Qi-stagnation constitution on postpartum depressive symptoms via early-pregnancy EPDS scores was significant (ab = 0.36, 95% Bootstrap CI: 0.19–0.57), with the direct effect remaining significant (c’ = 0.73, P = 0.014). This partial mediation model indicates that Qi-stagnation influences postpartum depressive symptoms through two concurrent pathways: a direct pathway, consistent with the emotional and psychological vulnerabilities associated with this constitution type (10), and an indirect pathway, in which Qi-stagnation first exacerbates depressive symptoms during early pregnancy, which in turn increase the risk of postpartum depressive symptoms. The indirect pathway may be explained by the fact that individuals with Qi-stagnation constitution exhibit poor emotional regulation and heightened stress reactivity (11, 17), making them more susceptible to accumulating psychological distress during the antenatal period. Such distress may persist into the postpartum period through physiological mechanisms (e.g., sustained hypothalamic-pituitary-adrenal axis dysregulation, postpartum hormonal fluctuations) (19) or through psychosocial mechanisms (e.g., insufficient social support, parenting stress) (20). Importantly, the persistence of a significant direct effect after accounting for the indirect pathway suggests that constitution-specific interventions—beyond the management of depressive symptoms alone—are needed to fully mitigate the risk of postpartum depressive symptoms in this vulnerable subgroup. The Balanced constitution significantly reduced postpartum depressive symptoms risk (OR = 0.38,95% CI: 0.24–0.61, P <0.001). After incorporating antenatal EPDS ≥ 9 into the multivariable model (Model 2, **Table 5**), the OR slightly decreased to 0.42 (95% CI: 0.26–0.68, P < 0.001), suggesting it may independently lower postpartum depressive symptoms risk via physiological or psychological mechanisms rather than just reducing prenatal depressive symptoms.

In addition, preterm birth—before 34 weeks of gestation—is associated with a more than fourfold higher risk of postpartum depressive symptoms (OR = 4.39, P = 0.007). Several mechanisms may explain this association. First, very preterm delivery is frequently accompanied by traumatic births, such as emergency cesarean or NICU (neonatal intensive care unit) admission, and those events themselves are known to raise the likelihood of psychological problems after delivery (21, 22). Second, mothers of very preterm infants undergo prolonged separation from their newborns and heightened caregiving burden, together with uncertainty about the infant’s prognosis, seem to lower maternal self- efficacy and to raise the depression risk (20). Third, the abrupt ending of normal pregnancy and the immediate need for intensive care in a highly stressful medical setting may prove too much for coping mechanisms of vulnerable women (especially if they already suffer from such issues as Qi stagnation constitution) (23).

These results highlight the importance of TCM constitution in postpartum depressive symptoms and the role of prenatal depressive symptoms as a precursor. Prevention and intervention for postpartum depressive symptoms should consider prenatal depressive symptoms, delivery gestational age, and constitution type, especially strengthening psychological support and constitution - based conditioning for Qi-stagnation - prone women, adopting a “TCM constitution - prenatal psychology - postpartum outcome” integrated intervention framework to leverage the advantages of integrated traditional and Western medicine.

### 4.3 Research significance and limitations

Through bootstrap mediation analysis, this study confirms that the severity of antenatal depressive symptoms serves as a partial mediator in the connection between Qi-stagnation constitution and postpartum depressive symptoms (indirect effect = 0.36, 95% CI: 0.19–0.57). This finding underscores the value of integrating TCM constitution identification with EPDS screening as a continuous measure in early pregnancy to identify high-risk women.

There are several limitations that should be taken into account. We did not gather information on psychosocial elements like social support, sleep quality, or negative life events, which could lead to residual confounding, even though TCM constitution is a comprehensive indicator that is closely linked to these aspects. Additionally, postpartum follow-up was limited to 14 days, which may not capture late-onset depressive symptoms. Future studies with extended follow-up and more comprehensive psychosocial assessments are needed to confirm our findings.

Clinically, we now suggest a two - step approach to screening at 10–14 weeks of gestation: TCM constitution analysis plus EPDS evaluation. For women with a Qi-stagnation constitution, we recommend a holistic approach that combines TCM-based conditioning with evidence-based psychological support, along with regular follow-ups to assess treatment effectiveness. Early detection combined with constitutionally appropriate treatment seems to lower the rate of perinatal depressive symptoms. Future studies need to assess the impact of these constitution-based interventions through randomized controlled trials.

## 5 Conclusion

TCM constitution, particularly Qi-stagnation and Balanced types, significantly influences the risk of perinatal depressive symptoms. Early screening and tailored interventions based on constitution may help reduce its incidence. Further research should explore the underlying mechanisms and evaluate the effectiveness of constitution-based interventions.

## Data Availability

The raw data supporting the conclusions of this article will be made available by the authors, without undue reservation.
